# Probable Acromioclavicular Joint Gout in Two Patients With Longstanding Gout: A Report of Two Cases

**DOI:** 10.1155/crrh/2289680

**Published:** 2026-10-28

**Authors:** Pheng Ann Kao, Leangsing Iv, Sotharith Bory, Xavier Chevalier

**Affiliations:** ^1^ Medicine A Ward Calmette Hospital Phnom Penh Cambodia; ^2^ Radiology Department Calmette Hospital Phnom Penh Cambodia; ^3^ Department of Rheumatology Henri Mondor Hospital Créteil France aphp.fr

**Keywords:** acromioclavicular joint, gout, hyperuricemia, tophaceous gout, ultrasonography

## Abstract

Acromioclavicular (AC) joint gout is uncommon and can be difficult to distinguish from infection or degenerative disease without synovial crystal confirmation. We describe two older men with longstanding, intermittently treated gout who presented with symptomatic AC‐joint abnormalities. A 74‐year‐old man had four months of bilateral AC‐joint swelling, peripheral tophi, hyperuricemia, erosive radiographic changes, and supportive ultrasonographic findings, including a double‐contour sign at the knee. A 94‐year‐old man had three months of right AC‐joint pain and swelling, hyperuricemia, radiographic irregularity, and ultrasonographic synovial changes with power Doppler activity. Neither patient had an accessible AC‐joint effusion for aspiration; therefore, crystal identification and bacterial culture were unavailable. The findings were compatible with probable AC‐joint gout, but infection could not be definitively excluded, especially in the second patient, whose C‐reactive protein was 74 mg/L. Colchicine and long‐term urate‐lowering therapy were prescribed; treatment details and standardized longitudinal outcomes were incompletely documented. These cases emphasize cautious diagnostic interpretation of unusual‐site gout without tissue confirmation.

## 1. Introduction

Gout is a crystal‐induced inflammatory arthropathy caused by deposition of monosodium urate (MSU) crystals in joints and periarticular tissues in the setting of sustained hyperuricemia. It most commonly affects peripheral joints, particularly the first metatarsophalangeal joint, although recurrent or longstanding disease may involve other appendicular sites. Axial involvement, including the spine and sacroiliac joints, is also recognized but is less common [1, 2]. Reported AC‐joint gout is rare and can mimic degenerative, infectious, or other inflammatory disorders of the shoulder region [3–8]. We report two patients with longstanding, inadequately treated gout and clinical and imaging findings compatible with probable AC‐joint gout, emphasizing the diagnostic limitations created by the absence of aspiratable joint fluid and the supportive role of ultrasonography.

## 2. Case Presentation

### 2.1. Case 1

A 74‐year‐old man was admitted in September 2024 with a 4‐month history of bilateral shoulder pain and swelling. The pain persisted at rest and frequently disturbed his sleep. He also reported pain in the fingers and toes. He denied fever or preceding trauma. He had a 15‐year history of gout and had used analgesics and urate‐lowering therapy (ULT) only intermittently during flares rather than continuously. He consumed at least five cans of beer daily and did not smoke. His medical history included hypertension, Type 2 diabetes mellitus, and chronic kidney disease with an estimated glomerular filtration rate of approximately 45 mL/min.

Examination showed bilateral AC‐joint swelling without limitation of shoulder movement. No clinically detectable AC‐joint effusion was present. Multiple subcutaneous tophi were present on the fingers and toes. Laboratory tests showed a white blood cell count of 8.6 G/L, hemoglobin 107 g/L, platelet count 225 G/L, C‐reactive protein 36 mg/L, creatinine 139 μmol/L, blood urea nitrogen 0.61 g/L, and serum uric acid 92 mg/L (9.2 mg/dL). Rheumatoid factor and anticitrullinated protein antibodies were negative.

Radiographs demonstrated soft‐tissue densities compatible with tophaceous deposits and punched‐out erosive changes in peripheral joints, together with abnormalities of both AC joints (Figures 1 and 2). Ultrasonography of the AC joints demonstrated synovial abnormalities with bright stippled foci/hyperechoic areas and erosive changes (Figure 3); a double‐contour sign was also identified at the right knee. Because there was no accessible AC‐joint effusion, arthrocentesis was not performed, and MSU crystals could not be confirmed by compensated polarized light microscopy. The overall clinical, laboratory, and imaging findings were considered compatible with chronic tophaceous gout and probable AC‐joint involvement. He received anti‐inflammatory treatment with colchicine, with nonsteroidal anti‐inflammatory therapy used cautiously in view of renal function, and was discharged on long‐term febuxostat as ULT. The patient remained under clinical follow‐up and was not lost to follow‐up. However, standardized longitudinal outcome measures, including serial pain scores, serum urate levels, inflammatory markers, repeat imaging, and recurrence assessments, were not systematically collected for this report because its primary focus was the diagnostic challenge rather than treatment efficacy or long‐term outcomes.

**FIGURE 1:**
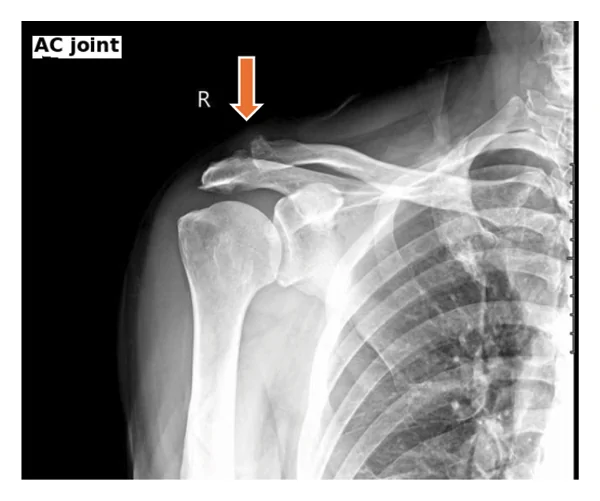
Case 1, right shoulder radiograph. The right acromioclavicular (AC) joint is labelled. Irregular and erosive changes are present and, in the clinical context, are supportive of probable AC‐joint gout.

**FIGURE 2:**
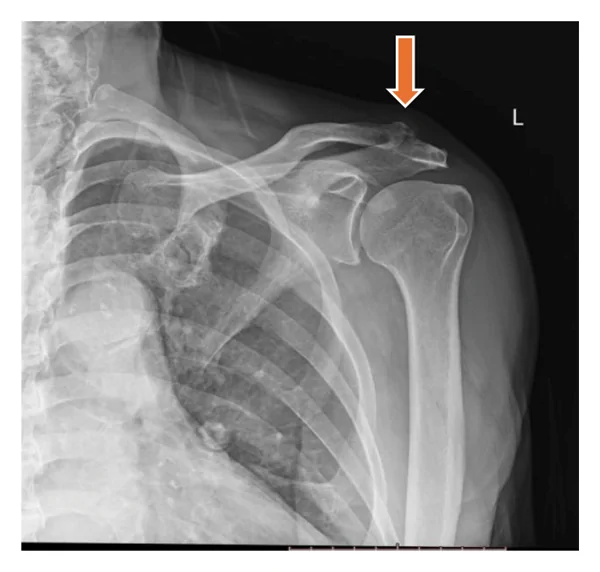
Case 1, left shoulder radiograph. The left AC joint is labelled, demonstrating structural abnormalities that, in the clinical context, are supportive of probable AC‐joint gout.

**FIGURE 3:**
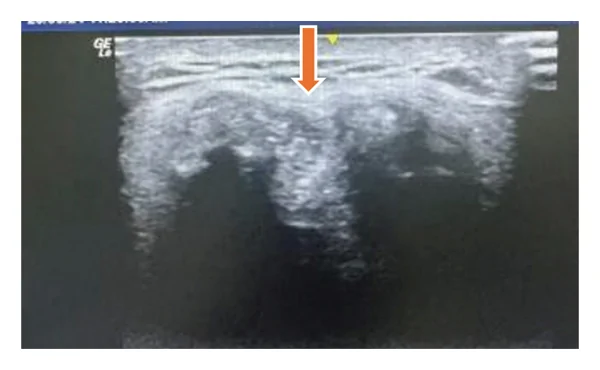
Case 1, representative AC‐joint ultrasonogram. The AC‐joint region is labelled, demonstrating synovial abnormality with bright stippled/hyperechoic foci. These findings provide supportive imaging evidence for probable AC‐joint gout but are not diagnostic in isolation.

### 2.2. Case 2

A 94‐year‐old man was admitted in November 2024 with a 3‐month history of right shoulder pain. The pain awakened him from sleep, was worse in the early morning, and persisted at rest. He reported no fever or other current joint involvement. He had experienced recurrent gout attacks since approximately 60 years of age and had used analgesics, nonsteroidal anti‐inflammatory drugs, and ULT only during attacks. He did not smoke and consumed approximately five cans of beer daily. He was receiving losartan 50 mg twice daily for hypertension.

Examination showed swelling and marked tenderness over the right AC joint without limitation of shoulder movement. Other joint and systemic examinations were unremarkable. Laboratory tests showed a white blood cell count of 8.44 G/L, hemoglobin 110 g/L, platelet count 304 G/L, C‐reactive protein 74 mg/L, creatinine 78 μmol/L, and serum uric acid 79 mg/L (7.9 mg/dL). Rheumatoid factor and anticitrullinated protein antibodies were negative.

Right shoulder radiography demonstrated AC‐joint irregularity (Figure 4). Ultrasonography showed synovial abnormality with hyperechoic areas and power Doppler activity (Figure 5). No accessible AC‐joint effusion was identified, and aspiration for MSU crystal examination or microbiological culture was not performed. The 3‐month symptom history, absence of reported fever, normal peripheral leukocyte count, longstanding recurrent gout, hyperuricemia, and supportive imaging favored probable AC‐joint gout. Nevertheless, the elevated C‐reactive protein (74 mg/L) and focal swelling and tenderness warranted consideration of infection, which could not be definitively excluded. The patient received colchicine and was discharged on long‐term febuxostat; clinical improvement was reported but not quantified. The patient remained under clinical follow‐up and was not lost to follow‐up. However, standardized longitudinal outcome measures were not systematically collected because the primary focus of this report was the diagnostic challenge rather than assessment of treatment efficacy or long‐term outcomes.

**FIGURE 4:**
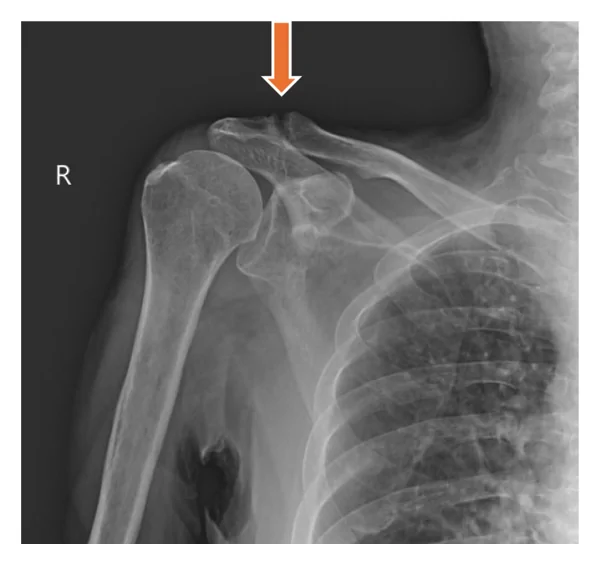
Case 2, right shoulder radiograph. The labelled AC joint demonstrates irregular structural change. In conjunction with the clinical history and other findings, the appearance is compatible with probable AC‐joint gout.

**FIGURE 5:**
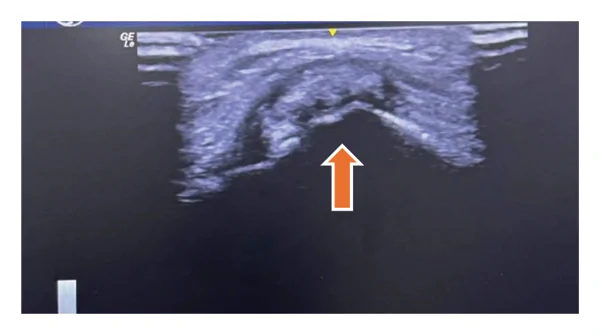
Case 2, right AC‐joint ultrasonogram. The labelled AC‐joint region demonstrates synovial abnormality with hyperechoic areas; power Doppler activity was documented during the examination. These findings are supportive of, but not specific for, probable AC‐joint gout.

## 3. Discussion

These two cases illustrate an uncommon anatomical presentation of chronic gout. Both patients had longstanding disease with intermittent rather than sustained ULT, persistent hyperuricemia, and features of substantial urate burden. In the first patient, clinically evident peripheral tophi and erosive disease provided additional evidence of chronic crystal deposition. Persistent serum urate above the saturation threshold promotes continued MSU deposition, while sustained urate lowering facilitates crystal dissolution and reduction of tophaceous burden. This pathophysiologic context provides a plausible explanation for involvement of an unusual site such as the AC joint, although the present two‐case report cannot establish a causal relationship.

AC‐joint gout is rarely reported. Previous reports have described monoarticular AC‐joint gout, bilateral AC‐joint involvement, cystic AC‐joint swelling, and gouty polyarthropathy involving the AC joint [3–8]. Some published cases occurred in association with factors such as lead exposure or medication use, whereas neither of our patients was treated with immunosuppressive drugs. The present cases are most notable for the combination of longstanding inadequately controlled gout, hyperuricemia, and imaging abnormalities at a symptomatic AC joint.

The principal diagnostic challenge is distinguishing probable AC‐joint gout from other causes of a swollen, painful AC joint. Direct identification of needle‐shaped, negatively birefringent MSU crystals remains the reference standard for confirming gout when aspirate or tophus material can be obtained. Neither patient had an accessible AC‐joint effusion; consequently, neither crystal examination nor bacterial culture was performed. In Case 2, the elevated CRP (74 mg/L), localized tenderness, and swelling made septic arthritis an important differential diagnosis. The prolonged course, lack of reported fever, normal leukocyte count, and longstanding gout favored a noninfectious explanation but did not exclude infection. In particular, older patients may lack fever or leukocytosis, and gout and infection can coexist. Synovitis and power Doppler activity are not specific for gout. If clinical concern for infection remains, prompt reassessment and image‐guided aspiration or other appropriate sampling should be considered whenever feasible. The absence of microbiological testing is a major limitation, and we do not claim that infection was excluded.

Ultrasonography can provide useful supportive evidence when aspiration is not possible. Recognized gout‐related ultrasound lesions include the double‐contour sign, tophi, aggregates/bright hyperechoic foci, and erosions [9, 10]. The 2015 ACR/EULAR gout classification criteria incorporate ultrasound evidence of urate deposition, particularly the double‐contour sign, as well as radiographic gout‐related erosion; however, classification criteria should not be treated as a substitute for crystal confirmation in an individual patient [9]. In the present report, ultrasound provided supportive, nonconfirmatory characterization of the symptomatic AC joints and demonstrated synovial abnormalities, hyperechoic foci/areas, erosive change, and, in the second patient, power Doppler activity. These findings should be interpreted together with the clinical history and other evidence of gout rather than as diagnostic of AC‐joint gout or as excluding infection.

Gout is not confined to peripheral joints. Axial gout involving the spine and sacroiliac joints has been reported and may present with back pain, radiculopathy, or other nonspecific manifestations [1, 2]. Awareness of both axial and unusual appendicular involvement is therefore important when evaluating patients with longstanding hyperuricemia or tophaceous gout.

Long‐term management should follow a treat‐to‐target strategy. The 2016 updated EULAR recommendations advise maintaining serum urate below 6 mg/dL (360 μmol/L) and below 5 mg/dL (300 μmol/L) in severe gout, including patients with a high crystal/tophaceous burden [11]. The 2020 ACR guideline strongly recommends ULT with serial serum urate monitoring to achieve and maintain a target below 6 mg/dL; it does not specify a lower numerical threshold for severe subgroups because of limited trial evidence, although lower urate concentrations may hasten tophus resolution [12]. For patients such as ours, continuous long‐term ULT and monitoring are therefore more appropriate than intermittent ULT use only during flares. In these cases, the documented medications were colchicine and febuxostat (with cautious NSAID use in Case 1); exact doses, treatment duration, dose escalation, flare prophylaxis, and serial renal function/urate monitoring could not be reconstructed from the available summary. These details should not be inferred from guideline recommendations. Case 1 had reduced kidney function (eGFR approximately 45 mL/min), reinforcing the need for individualized medication safety assessment.

Several limitations constrain interpretation. Neither AC joint was crystal‐confirmed or sampled for microbiology, nor dual‐energy computed tomography was not performed. Ultrasound images were obtained during routine care and varied in quality; AC‐joint hyperechoic foci and erosions are not independently diagnostic. Although both patients remained under clinical follow‐up and neither was lost to follow‐up, standardized longitudinal assessments, including serial pain scores, serum urate and CRP measurements, repeat imaging, and systematic recurrence assessment, were not performed for this report. This reflects the primary objective of the report, which was to describe the diagnostic challenge of probable AC‐joint gout rather than to evaluate treatment efficacy or long‐term outcomes. Medication doses, durations, titration schedules, flare prophylaxis, and serial safety monitoring could not be reliably reconstructed from the clinical summaries. Therefore, no conclusions regarding sustained therapeutic response, recurrence, serum‐urate target achievement, or long‐term disease control can be drawn.

## 4. Conclusion

In patients with longstanding gout, focal AC‐joint pain or swelling may reflect probable gout involvement, especially when supported by tophi or imaging evidence of urate deposition elsewhere. Radiography and ultrasonography can strengthen clinical suspicion but do not establish crystal‐proven AC‐joint gout. When aspiration is not feasible, clinicians should acknowledge residual diagnostic uncertainty, particularly the inability to definitively exclude septic arthritis. These cases underscore the importance of integrating clinical history, imaging, and the need for microbiological evaluation when infection is suspected.

## Author Contributions

Pheng Ann Kao: conceptualization, patient care, data collection, literature review, and writing of the original draft.

Leangsing Iv: radiologic and ultrasonographic assessment, interpretation of imaging findings, and review of the manuscript.

Sotharith Bory: clinical assessment, patient care, and critical review and editing of the manuscript.

Xavier Chevalier: supervision, rheumatologic expertise, interpretation of the clinical findings, and critical review and editing of the manuscript.

## Funding

The authors received no specific funding for this work.

## Disclosure

All authors reviewed and approved the final version of the manuscript.

## Ethics Statement

Ethical approval was not required for this case report according to local institutional requirements. Written informed consent for publication was obtained from both patients.

## Consent

Written informed consent was obtained from both patients for the publication of their anonymized clinical information and accompanying images.

## Conflicts of Interest

The authors declare no conflicts of interest.

## Data Availability

Data sharing is not applicable to this article, as no datasets were generated or analyzed during the preparation of this case report.
